# N_2_ fixation dominates nitrogen cycling in a mangrove fiddler crab holobiont

**DOI:** 10.1038/s41598-020-70834-0

**Published:** 2020-08-18

**Authors:** Mindaugas Zilius, Stefano Bonaglia, Elias Broman, Vitor Gonsalez Chiozzini, Aurelija Samuiloviene, Francisco J. A. Nascimento, Ulisse Cardini, Marco Bartoli

**Affiliations:** 1 Marine Research Institute, Klaipėda University, Klaipeda, Lithuania; 2 Department of Life Sciences and Biotechnology, University of Ferrara, Ferrara, Italy; 3 Department of Ecology, Environment and Plant Sciences, Stockholm University, Stockholm, Sweden; 4 Department of Biology, University of Southern Denmark, Odense, Denmark; 5 Baltic Sea Centre, Stockholm University, Stockholm, Sweden; 6 Oceanographic Institute, University of São Paulo, São Paulo, Brazil; 7 Integrative Marine Ecology Department, Stazione Zoologica Anton Dohrn, National Institute of Marine Biology, Ecology and Biotechnology, Napoli, Italy; 8 Department of Chemistry, Life Science and Environmental Sustainability, University of Parma, Parma, Italy

**Keywords:** Microbiology, Biogeochemistry, Ecology

## Abstract

Mangrove forests are among the most productive and diverse ecosystems on the planet, despite limited nitrogen (N) availability. Under such conditions, animal-microbe associations (holobionts) are often key to ecosystem functioning. Here, we investigated the role of fiddler crabs and their carapace-associated microbial biofilm as hotspots of microbial N transformations and sources of N within the mangrove ecosystem. 16S rRNA gene and metagenomic sequencing provided evidence of a microbial biofilm dominated by Cyanobacteria, Alphaproteobacteria, Actinobacteria, and Bacteroidota with a community encoding both aerobic and anaerobic pathways of the N cycle. Dinitrogen (N_2_) fixation was among the most commonly predicted process. Net N fluxes between the biofilm-covered crabs and the water and microbial N transformation rates in suspended biofilm slurries portray these holobionts as a net N_2_ sink, with N_2_ fixation exceeding N losses, and as a significant source of ammonium and dissolved organic N to the surrounding environment. N stable isotope natural abundances of fiddler crab carapace-associated biofilms were within the range expected for fixed N, further suggesting active microbial N_2_ fixation. These results extend our knowledge on the diversity of invertebrate-microbe associations, and provide a clear example of how animal microbiota can mediate a plethora of essential biogeochemical processes in mangrove ecosystems.

## Introduction

Among coastal ecosystems, mangrove forests are of great importance as they account for three quarters of the tropical coastline and provide different ecosystem services^1,2^. Mangrove ecosystems generally act as a net sink of carbon, although they release organic matter to the sea in the form of dissolved refractory macromolecules, leaves, branches and other debris^3,4^. In pristine environments, mangroves are among the most productive ecosystems on the planet, despite growing in tropical waters that are often nutrient depleted^5^. The refractory nature of the organic matter produced and retained in mangroves can slow the recycling of nutrients, particularly of nitrogen (N)^3,6^. Nitrogen limitation in such systems may be overcome by microbial dinitrogen (N_2_) fixation when combined with high rates of bioturbation by macrofauna^7,8^.

Bioturbation by macrofauna affect N availability and multiple N-related microbial processes through sediment reworking, burrow construction and bioirrigation, feeding and excretion^9^. Macrofauna mix old and fresh organic matter, extend oxic–anoxic sediment interfaces, increase the availability of energy-yielding electron acceptors and increase N turnover via direct excretion^10,11^. Thus, macrofauna may alleviate N limitation by priming the remineralization of refractory N, reducing plants-microbe competition^12,13^. Such activity ultimately promotes N-recycling, plant assimilation and high N retention, as well as favours it loss by stimulating coupled nitrification and denitrification^14^.

Mangrove sediments are highly bioturbated by decapods such as crabs^15^. Crab populations continuously rework sediment by constructing burrows, creating new niches, transporting or selectively grazing on sediment microbial communities^15–18^. In addition, crabs can affect organic matter turnover by assimilating leaves and producing finely fragmented faeces, or by carrying them into their burrows^19,20^. Therefore, crabs are considered important ecosystem engineers shaping biogeochemical processes in intertidal muddy banks of mangroves^21–23^. In contrast to burrowing polychaetes or amphipods, the abundant Ocipodid crabs, mainly represented by fiddler crabs, do not permanently ventilate their burrows. These crabs may temporarily leave their burrows for surface activities^18^, or otherwise plug their burrow entrance during tidal inundation in order to trap air^24^. A recent study by Cuellar-Gempeler and Leibold^17^ showed that these crabs can be associated with a diverse microbial community, either on their carapace or in their gut.

The exoskeleton of living animals, such as shells or carapaces, offers a habitat for microbial biofilms which are actively involved in different N-cycling pathways such as nitrification, denitrification and dissimilatory nitrate reduction to ammonium (DNRA)^25–30^. Colonizing the carapace of crabs may be advantageous for specific bacteria, because of host activities such as respiration, excretion, feeding and horizontal and vertical migrations^31^. However, the ecological interactions between fiddler crabs and bacteria, their regulation and significance as well as their implications at scales spanning from the single individual to the ecosystem are not well understood^16,32^.

Prior manipulative laboratory experiments have explored the density-dependent effects of macrofauna on selected N processes (e.g., *Corophium* density vs. sedimentary denitrification rates^33^). So far studies have addressed the three dimensional redox environment created by active burrowers, supported by microelectrode^34^ and later by planar optode measurements^35^. More recently, the increasing synergy between biogeochemical approaches and molecular techniques have opened new avenues of research on N-cycling at the scale of a single macrofauna individual and its associated microbial community, i.e., the holobiont^36–38^.

Here, we hypothesized that the fiddler crab holobiont (here *Leptuca thayeri*) may represent a N-cycling hotspot and specifically a N source for bioturbated mangrove ecosystems. To assess this hypothesis, we integrated molecular techniques and N-related biogeochemical measurements on crabs collected from a protected Brazilian mangrove system with a low inorganic N background. Such habitat is representative of the most pristine mangrove forests worldwide^39^. 16S rRNA gene amplicon and metagenomic sequencing allowed to explore the taxonomic and functional diversity of the crabs’ carapace microbiota. This was complemented by qPCR assays that allowed to quantify the abundance of relevant functional genes regulating nitrate (NO_3_^−^) production and consumption. Additionally, net N fluxes and ^15^N probing experiments were used to quantify microbial N transformations and animal excretion, allowing for a mechanistic understanding of holobiont N-cycling.

## Results

### Systematic diversity of the crab’s carapace microbiota

**Figure 1:**
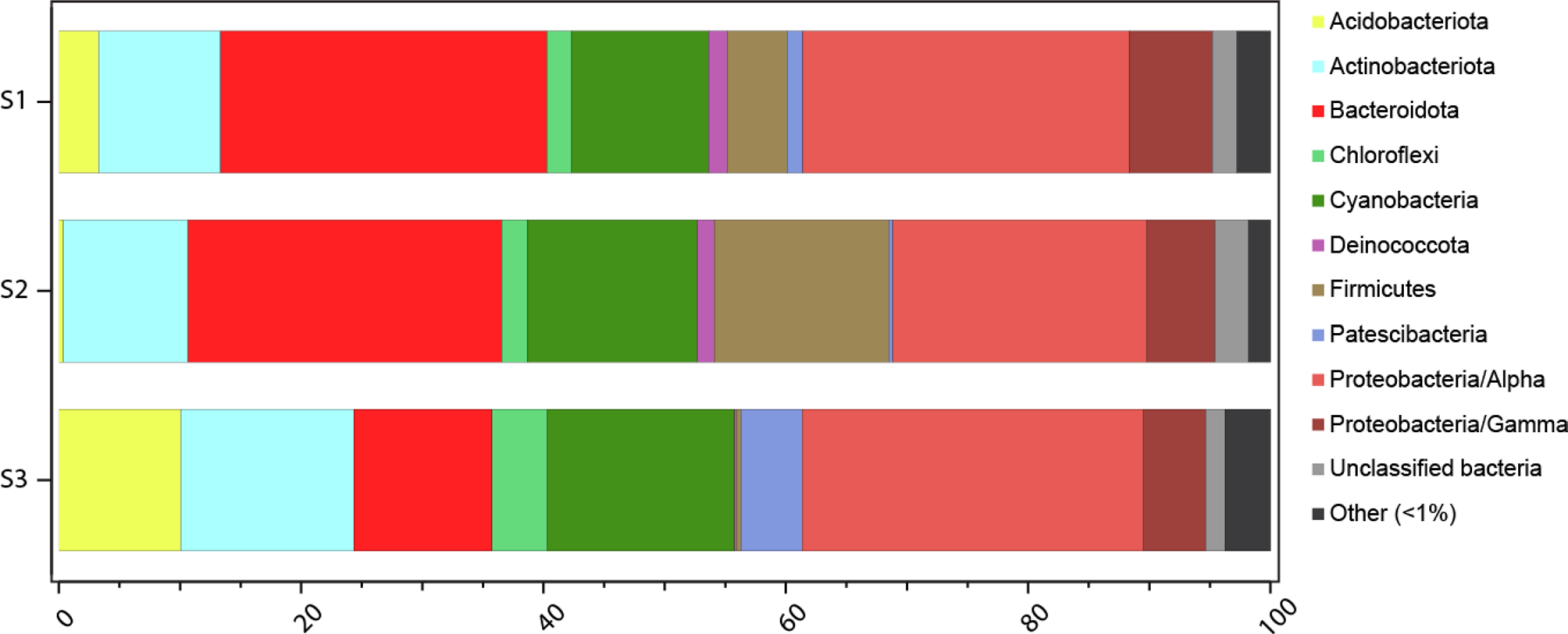
Systematic diversity of the crab’s carapace microbiota: Relative abundance of bacterial phyla and Proteobacteria classes obtained by 16S rRNA gene sequencing. Taxa contributing < 1% are not shown.

The 16S rRNA gene amplicon sequencing with subsequent DADA2 analysis yielded 1913 amplicon sequence variants (ASVs). Shannon’s H alpha diversity in the 16S rRNA gene data was 5.5 ± 0.1 (mean ± standard error, n = 3). The analysis showed that the dominating prokaryotic phyla on the biofilm-covered carapace were Alphaproteobacteria (25.3 ± 2.2%) and Bacteroidota (21.4 ± 5.0%) followed by Cyanobacteria (13.6 ± 1.2%) and Actinobacteria (11.5 ± 1.4%) (Fig. 1). Most of these sequences were attributed to the Cyanobacteria order Cyanobacteriales, followed by Alphaproteobacteria orders Sphingomonadales and Rhodobacterales, and the Bacteroidota order Flavobacteriales (Supplementary Fig. 1, Supplementary Information 1). The ASVs with the highest relative abundance belonged to the Cyanobacteria genus *Geitlerinema* PCC-7105 (12.3 ± 1.1%; Supplementary Fig. 1). The top Bacteroidota ASVs included unclassified *Flavobacteriaceae* sequences (6.6 ± 1.7%) and the genus *Hoppeia* (5.2 ± 1.5%) belonging to the same family. Top Alphaproteobacteria genera included *Erythrobacter* and *Paracoccus* (8.0 ± 3.0 and 2.5 ± 0.4%, respectively). Shotgun metagenomic sequencing yielded on average 9,433,482 sequences (R1 and R2) that could be classified for microbial taxa based on functional genes classified against NCBI NR and imported into MEGAN. The analysis returned results similar to the ones obtained by 16S rRNA gene amplicon sequencing, with a community dominated by Alphaproteobacteria, Cyanobacteria, Bacteroidetes (Bacteroidota in the SILVA database), and Actinobacteria, despite the comparison between these two datasets presents limitations and many bacteria remain unclassified in the metagenomic database.

### N-cycling functional diversity of the crab’s carapace microbiome

**Figure 2:**
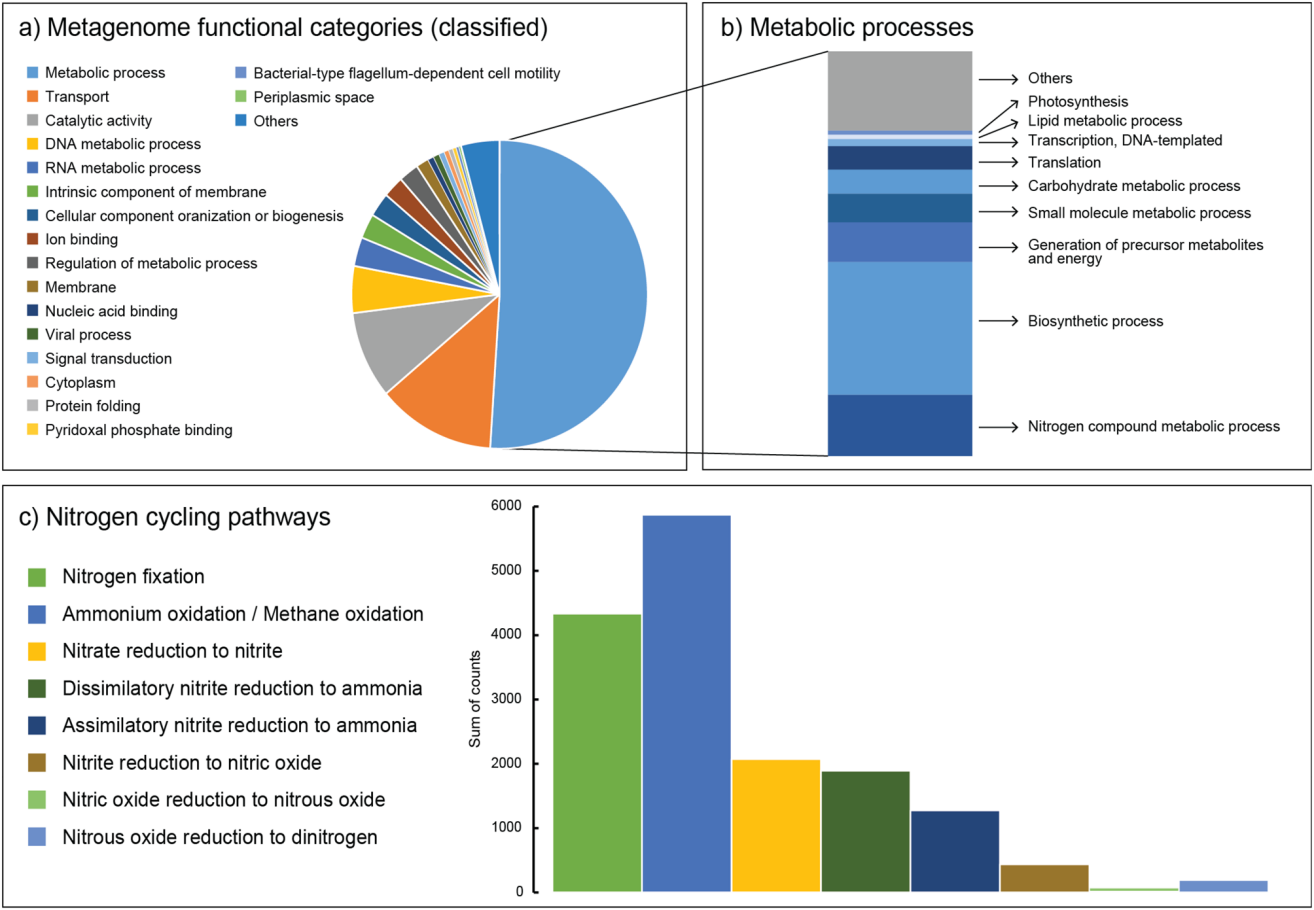
Functional profiling of the crab’s carapace microbiota using metagenomics: (**a**) Gene Ontology functional categories of metagenome sequences (%) that constituted the majority of the metagenomic data (45.3% classified reads) with (**b**) a breakdown of the subcategories (%) among the classified metabolic processes and (**c**) metabolic processes of the nitrogen cycle (5% of the “Nitrogen compound metabolic process” subcategory). Pathways consist of the sum of counts for related proteins. The analysis was limited to functional categories > 0.1%.

Most of the metagenome reads were attributed to the Gene Ontology categories: metabolic processes, transport, and catalytic activity (Fig. 2a). Among metabolic processes, the main subcategories were those related to aerobic respiration, central carbon cycling, peptidases, and housekeeping genes, but also pathways involving N compounds (Fig. 2b). Browsing of the proteins involved in N-cycling (according to the KEGG nitrogen metabolism reference pathway: https://www.genome.jp/kegg-bin/show_pathway?map00910) revealed that the microbial community was capable of all N-cycling pathways, including N_2_ fixation, ammonium (NH_4_^+^) oxidation (and potentially methane oxidation), NO_3_^−^ reduction to nitrites (NO_2_^−^) and assimilatory NO_2_^−^ reduction to NH_4_^+^, dissimilatory NO_2_^−^ reduction to NH_4_^+^, NO_2_^−^ reduction to nitric oxide (NO) and subsequent reduction to nitrous oxide (N_2_O) and to N_2_ (Fig. 2c). The studied N-cycling pathways represented 0.32% of all the classified proteins, 0.75% of the “Metabolic processes” category, and 5% of the “Nitrogen compound metabolic process” subcategory. A full list of all classified proteins is provided in Supplementary Information 2. Unclassified sequences attributed to the *nifH/frxC* protein family were included in the N_2_ fixation category (sequences classified to specific *frxC* family proteins were not included). Major prokaryotic groups that could be taxonomically classified and attributed to these processes were indicated to be Alphaproteobacteria (24% of all N-cycling related sequences), Cyanobacteria (20%), Bacteroidetes (9%), Actinobacteria (6%), Chloroflexi (2%) and Gammaproteobacteria (2%) (Supplementary Information 2). In more detail, these phyla constituted e.g. taxonomic orders Sphingomonadales, Rhodobacterales, Flavobacteriales, and Nostocales (Supplementary Information 2). Our identified prokaryotes harboured a large array of metabolic features, carrying marker genes for up to 5 different N-cycling pathways (e.g., Alphaproteobacteria). For example, NO_3_^−^ reduction pathways in biofilms were carried out by Alphaproteobacteria, Actinobacteria, Bacteroidetes, Cyanobacteria, Chloroflexi and Gammaproteobacteria, as shown in the metagenome data (Supplementary Information 2).

### Abundance of key N-cycling genes

qPCR assays confirmed the genetic potential of the microbial community to produce or reduce NO_3_^−^ on the carapace. Functional *nrfA* and *nirS/K* genes, involved in NO_2_^−^ reduction to NH_4_^+^ and NO were simultaneously present on the biofilm-covered carapace (Table 1). The relative abundance of biofilm associated *nirS* and *nrfA* were 6.7 ± 3.1 × 10^–5^ and 10.5 × 10^–5^ per 16S rRNA gene copy, respectively. Contrarily, NH_4_^+^ oxidation to NO_2_^−^ had considerably lower capacity, contributed solely by archaea (2.1 ± 0.2 × 10^–6^ per 16S rRNA gene copy). Bacterial *amoA* was not detected on carapace by qPCR.

**Table 1:** Abundance of functional marker genes associated with N-cycling on the fiddler crab carapace.

Functional gene	Copy number in sample	
*nirS*	1.53 ± 0.13 × 10^3^	*n* = 3
*nirK*	0.14 × 10^3^	*n* = 3
*nrf*	2.42 × 10^3^	*n* = 3
*amoA-archaeal*	0.08 ± 0.04 × 10^3^	*n* = 3
*amoA-bacterial*	Not detected	*n* = 3

Note that *nirK* and *nrfA* was detected only in two of three samples. Average and standard errors are given.

### Net N fluxes associated with fiddler crab holobiont

Biofilm-covered crabs actively released NH_4_^+^ and dissolved organic nitrogen (DON) which corresponded to 34% and 57% of total dissolved N production (DIN + DON) in the microcosms with crabs, respectively (Table 2). Net production of NO_3_^−^ and NO_2_^−^ was quantitatively less important, comprising together < 9% of total dissolved N production. Net N_2_ fluxes in the microcosms were negative, indicating the dominance of N_2_ fixation over denitrification.

**Table 2:** Nitrogen fluxes associated with fiddler crab individuals incubated in microcosms.

Measure	Rates µmol N g_dw_^−1^ crab d^−1^	
N_2_	− 12.98 ± 2.93	*n* = 5
NH_4_^+^	1.10 ± 0.21	*n* = 5
NO_2_^-^	0.07 ± 0.01	*n* = 5
NO_3_^-^	0.21 ± 0.03	*n* = 5
DON	1.81 ± 2.17	*n* = 5
TDN	3.18 ± 2.14	*n* = 5

Average and standard errors are given. Total dissolved nitrogen was calculated as sum of NH_4_^+^, NO_2_^-^, NO_3_^-^ and DON.

### Microbial NO_3_^−^ reduction and production in the biofilm

**Figure 3:**
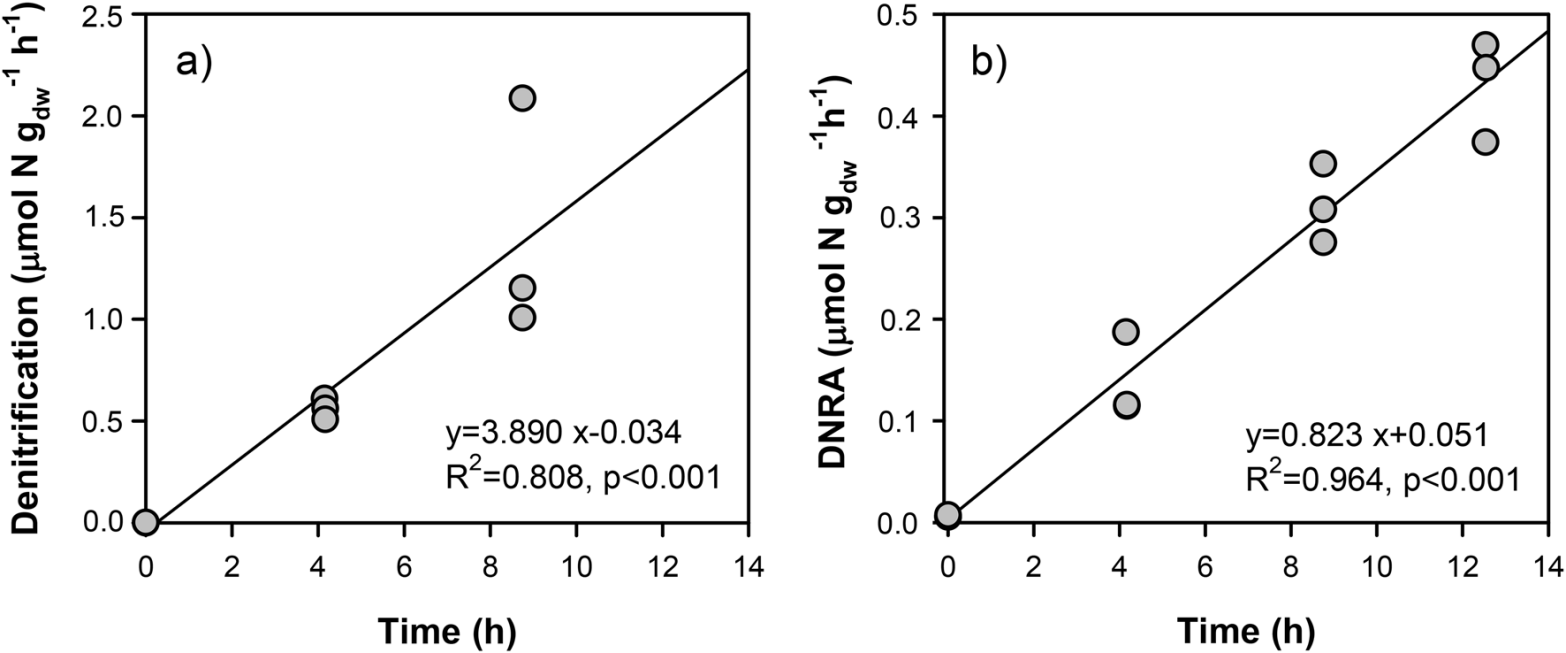
Nitrate reduction processes in crab carapace biofilm slurries: (**a**) production of N_2_ via denitrification and (**b**) production of NH_4_^+^ via dissimilative nitrate reduction to ammonia (DNRA). Biofilm slurries were amended with ^15^NO_3_^−^ and incubated over a period of 12 h, and represent potential rates. Note that production of N_2_ is not shown for the last time point as it exceeded the measuring rage of the instrument. Both potential rates are expresses as micromoles of N per g dry weight of suspended biofilm.

Anoxic slurry incubations of biofilms collected from multiple crab carapaces revealed that the dominant process was denitrification with 3.89 ± 0.72 µmol N g_dw_^−1^ biofilm d^−1^, while DNRA was considerably lower (0.82 ± 0.05 µmol N g_dw_^−1^ biofilm d^−1^) (Fig. 3). Anammox was a negligible process in the biofilm, as ^29^N_2_ production was always below the detection limit (data not shown). In the oxic biofilm slurries, NO_3_^−^ increased from 6.4 to 20.6 µmol N l^−1^, which corresponded to 0.56 ± 0.01 µmol N g_dw_^−1^ biofilm d^−1^ of potential nitrification rate.

### Natural abundance of stable isotopes

**Figure 4:**
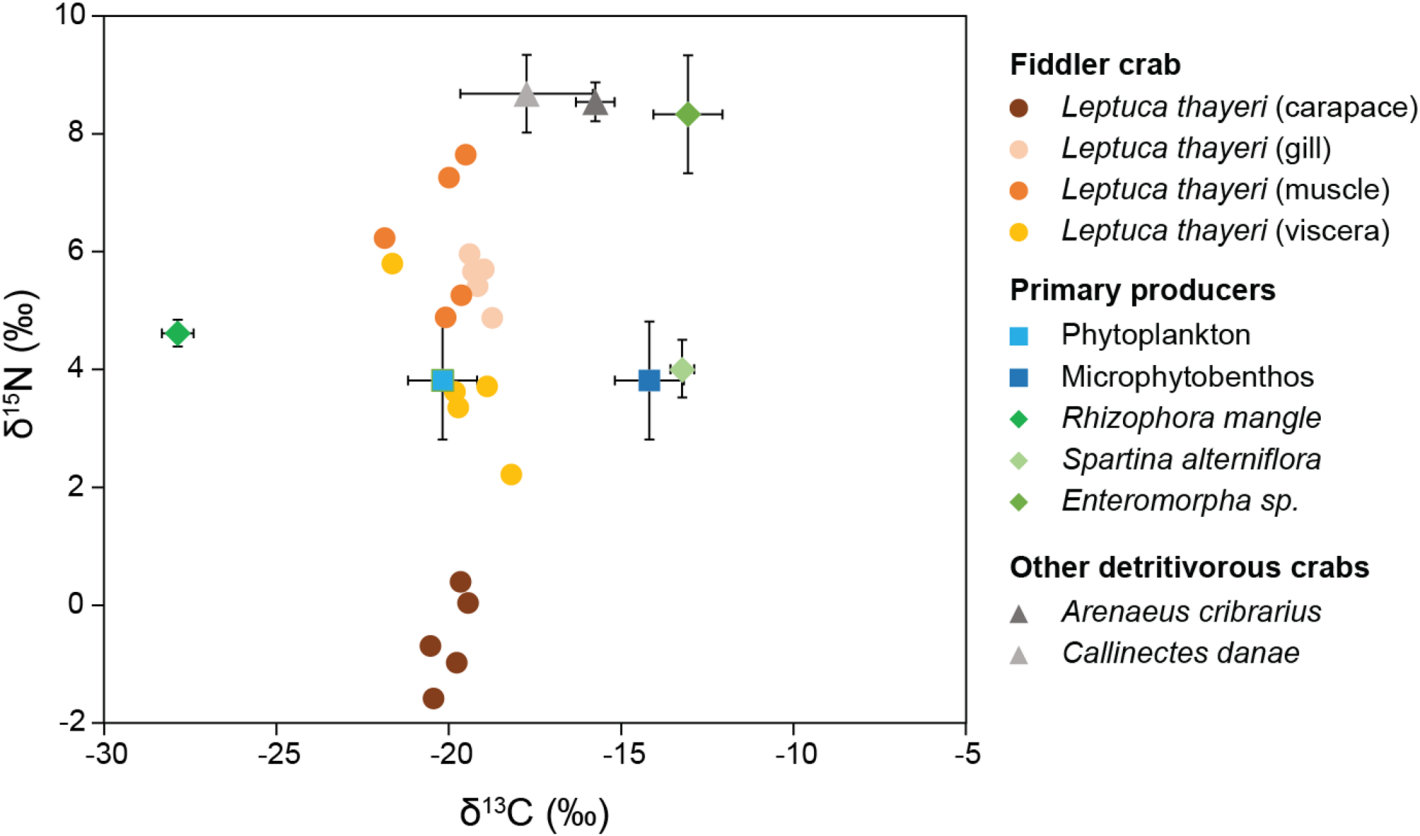
Signature of N_2_ fixation in crab’s carapace biofilms stable isotope ratios: biplot of the natural abundance of ^13^C and ^15^N isotopes for different samples (carapace, gill, muscle, viscera) from the fiddler crab (*Leptuca thayeri*, n = 5), and for different primary producers (as potential sources of detritus) and other detritivores crab species from the Cananéia estuarine system (data from Nagata et al.^40^).

The δ^13^C values were similar among all crab tissues, while δ^15^N values varied among samples with lowest values found in carapace biofilm samples (Fig. 4, Supplementary Information 3). Plotting natural abundances in a biplot together with values recorded in the literature for multiple primary producers and other crab species from the same site (data from Nagata et al.^40^) shows that crab tissues (gill, muscle and viscera) are depleted with regards to ^15^N as compared to other crab species (Fig. 4).

## Discussion

**Figure 5:**
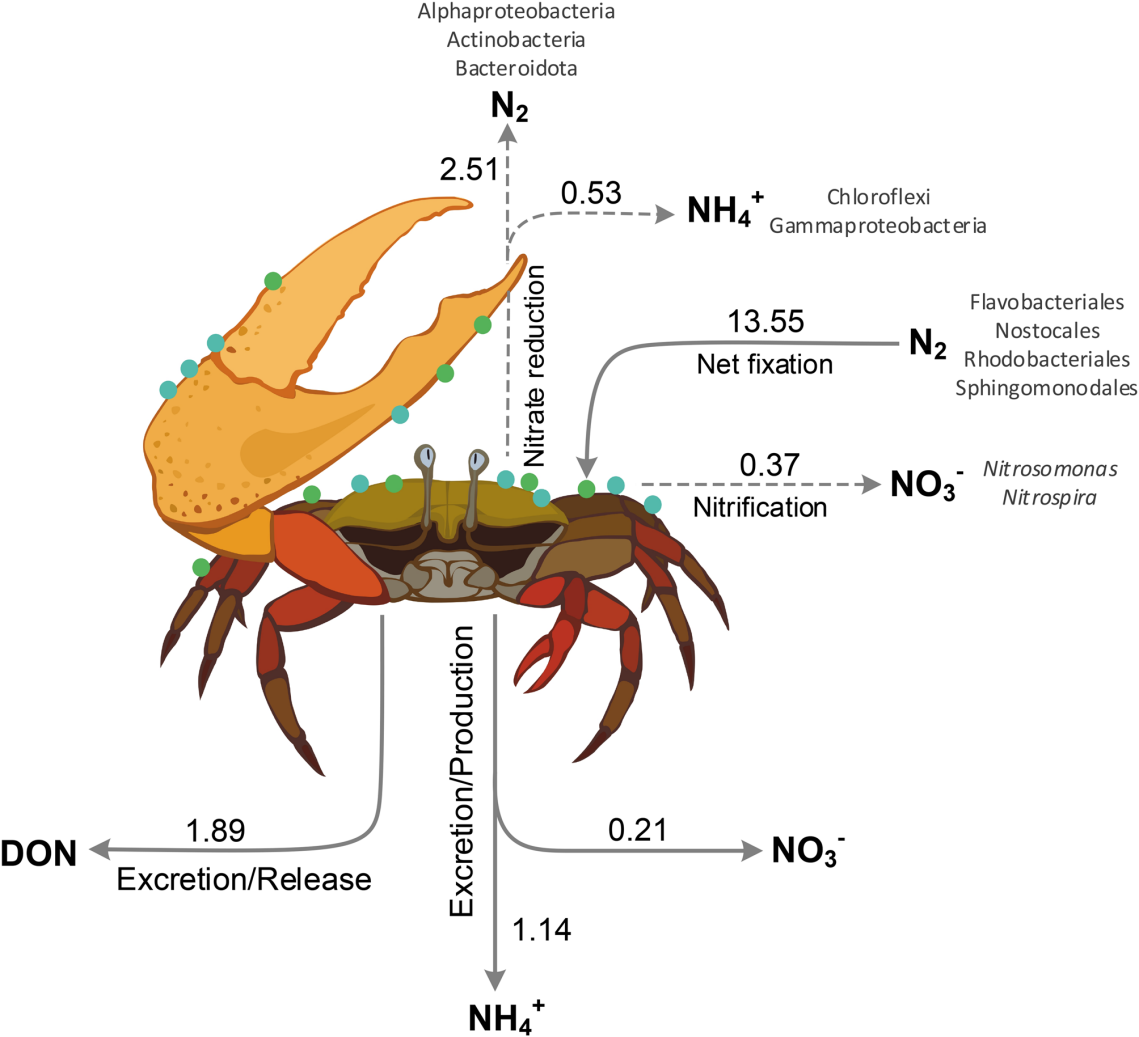
Flowchart of N-cycling by the fiddler crab holobiont: all fluxes were obtained combining data from incubations of single crab holobiont (in situ rates, solid lines) and suspended biofilm (potential rates, dashed lines). Note that reported rates differ from those in Table 2 as they were normalized per mean dry weight of crab’s biofilm or per mean dry weight of incubated fiddler crab and are expressed as µmol N crab^−1^ d^−1^. The main taxonomic groups involved in N-cycling as indicated by metagenomics analyses are provided. Drawing by V. Gasiūnaitė.

Using multiple lines of evidence integrating biogeochemical measurements, stable isotope probing, 16S rRNA gene and metagenomic sequencing and qPCR of functional genes, we constructed a flowchart of holobiont N-cycling (Fig. 5). Combining evidence from potential and measured fluxes with the key microbial players presumably responsible of N transformations within the biofilm, we show that fiddler crab holobionts are hotspots of benthic N-cycling, acting as relevant sources of fixed N to their surrounding environment.

### The crab microbiota fix atmospheric N_2_

The highest measured rates were attributed to negative N_2_ fluxes, suggesting N_2_ fixation as the dominant process. N_2_ fixation was also one of the most dominant metabolic processes compared to other N-cycling pathways within the metagenome, and depleted ^15^N stable isotope signatures further point at N_2_ fixation as a relevant process within the biofilm. Dinitrogen fixation is a common process in mangrove ecosystems, where the highest activities are found associated with the mangrove rhizosphere^39^. The presence of N_2_ fixing prokaryotes in the mangrove rhizosphere is often explained by a mutualistic relationship between the bacteria and the plant, with roots exuding dissolved carbon required for diazotrophic growth^41^. Similarly, although it remains speculative whether fiddler crabs take advantage of N_2_ fixers residing on their carapace, the depleted ^15^N signatures in their tissues compared to other detritivores crab species from the same site may indicate a nutritional relationship between the fiddler crab host and its microbiota. Additionally, the alpha diversity of the crab biofilm prokaryotic community (Shannon’s H ~ 5.5) was within the lower range of prokaryotic diversity reported in previous studies focusing on sediments^42,43^, suggesting the occurrence of selection processes on the crab’s carapace.

Biofilm-covered fiddler crabs were collected from an open muddy bank near a tidal creek, where sunlight stimulates the growth of unique phototrophic microorganisms, different from those found landward^44^. These fiddler crabs constantly migrate between burrows and the sediment surface and such vertical and horizontal migrations across contrasting environmental gradients (light regime, salinity, redox conditions, nutrients, organic matter) have the potential to create strong selective pressures on its biofilm-associated prokaryotes, although prior studies found that the fiddler crabs’ carapace was colonized by multiple microbial pools from the burrow walls^17,18^.

The most represented taxa within the biofilm was the Cyanobacteria genus *Geitlerinema*, which dominated the community with ~ 12% of all 16S rRNA gene sequences (see Supplementary Fig. 1) suggesting an important role within the assemblage. *Geitlerinema* contains biofilm-forming species that are capable of photosynthetic anoxygenic N_2_ fixation^45^. Due to its environmental plasticity, *Geitlerinema* spp. photosystem machinery may reduce oxygen production allowing oxygen-sensitive processes such as N_2_ fixation to operate in its cell or in neighbouring bacteria^45^. Other potential N_2_ fixers in our dataset were affiliated to well-known diazotrophic bacterial taxa like Sphingomonadales, Rhodobacteriales and Flavobacteriales. For example, *Erythrobacter* (the top alphaproteobacterial ASVs, Supplementary Fig. 1) is suspected to be diazotrophic^46^ and forms facilitative consortia with N_2_-fixing cyanobacteria^47^. Similarly, on the crabs’ carapace, a N_2_-fixing consortium (Fig. 5) might represent an association arranged along environmental gradients within the biofilm (e.g. light, oxygen) and whose strategy is to cooperate in order to enhance diazotrophic activity under N-limiting conditions^48,49^. The dominance of *Geitlerinema* spp. might be ascribed to its metabolic plasticity allowing it to cope with both high sulphide concentrations, which may build-up in clogged burrows, and air exposure when the crab leaves to the surface^45^.

### Nitrogen is both assimilated and lost

Nitrification (i.e. the recycling of N through oxidation of NH_4_^+^ to NO_3_^−^) doesn’t appear to play an important role on the crab’s carapace (Fig. 5). Accordingly, nitrifying bacteria affiliated to *Nitrospira* and *Nitrosomonas* represented only a minor fraction of the biofilm bacterial community. The low abundance of these microbial taxa is also a common feature across different benthic environments^50,51^. Sequences encoding proteins such as *amoA* and likely *pmoA*, responsible for NH_4_^+^ and methane oxidation, were present in the metagenome. However, it is difficult to discriminate the relevance of nitrification from the metagenomic dataset, as both monooxygenases are membrane-bound and evolutionary-related enzymes, thus frequently annotated together^52^. Additionally, our measured potential rates of nitrification were low, further indicating limited nitrification (Fig. 5).

Sequences encoding key enzymes for both assimilative and dissimilative (denitrification and DNRA) NO_3_^−^ reduction process were detected in our metagenome, indicating the metabolic potential for both these processes to occur on the carapace. Though DNRA genetic potential was quantitatively similar to that of denitrification, process measurements indicated a much higher expression of the latter, and denitrification was a dominant pathway of NO_3_^−^ reduction in the biofilm slurries (Fig. 5). Nevertheless, despite the high potential denitrification rates found in our incubations under anaerobic conditions, the metabolic capacity of denitrifiers is likely limited by low NO_3_^−^ background concentrations in the surrounding water (NO_3_^−^ < 0.5 µM). A previous study showed that NO_3_^−^ produced during nitrification can partly support denitrification inside the biofilm^28^. However, in our study nitrification and excretion by the fiddler crab holobiont could only support 8–15% of denitrification potential. Furthermore, the high potential of assimilatory NO_2_^−^ reduction to NH_4_^+^ in the biofilm likely promotes competition for NO_3_^−^ between the different microbial populations. Therefore, the predominance of potential denitrification over DNRA in our biofilm slurry incubations might be explained by the C:N ratio or by NO_2_^−^ concentrations^53^.

The major groups likely involved in dissimilative processes in our samples were Alphaproteobacteria, Actinobacteria, Bacteroidota, Chloroflexi and Gammaproteobacteria, taxa which frequently harbour these proteins in the marine environment^54^. The genuses *Janibacter* (*Intrasporangiaceae*) and *Aquimarina* (*Flavobacteriaceae*) were highly represented in the crab’s microbiota and both have the metabolic capacity to reduce NO_3_^−^^55,56^. Most identified bacteria possess all four genes (*nar/nap*, *nir*, *nor* and *nos*) which allow for complete NO_3_^−^ reduction to N_2_ as the end product^57^. The genus *Blastomonas* (Sphingomonadales), also highly represented, include members known for their chemotrophic lifestyle, capable of using NO_3_^−^ and its reduction products as electron acceptors^51,58–61^. Furthermore, the phylogenetic assignment of *nar* and *nir* genes indicates that the cyanobacterium *Geitlerinema* spp. may be involved in both N_2_ fixation and dissimilative NO_3_^−^ reduction. On the other hand, the major groups containing the array of genes NapC/NirT/Nrf (therein *nrf*A), responsible for DNRA, were assigned primarily to Chloroflexi (Anaerolineae) and to unclassified Gammaproteobacteria.

The transcription of most marker genes encoding for NO_3_^−^ reduction and its derivatives typically occurs under low oxygen conditions^62^, which we may also expect within the crab’s biofilm. However, temporal oxygen fluctuation on the carapace is inevitable when the host leaves its burrow to the surface and it is exposed to air and oxygen production via photosynthesis by associated phototrophs (e.g. Nostocales). Oxygen rise should supress the metabolic capacity of anaerobic bacteria, and especially of those that are respiring NO_3_^−^. However, Marchant et al.^63^ demonstrated that in dynamic environments with strong oxygen oscillations such as permeable sediments subject to daily tidal inundations, the transcription of denitrification genes happens both under oxic and anoxic conditions, suggesting that in the short time (hours) terminal reductases are not immediately suppressed and the electron flow continues. The occurrence of anoxic niches within the biofilm would further allow the microbial community to physiologically dispose of excess reducing equivalents^63,64^. Aerobic denitrification, on the other hand, is unlikely to occur under these conditions as indicated by the low number of reads of the key marker gene—periplasmic nitrate reductase (*nap*; review by^65^)—in our samples. Similarly, anammox appeared to be a negligible process within the biofilm, probably being suppressed by the fluctuating environmental conditions (temperature, O_2_, and nutrient concentrations) experienced by the crab holobionts, which are unfavourable to slow-growing anammox bacteria^66^. This is also the case in mangrove sediments where the contribution of anammox to N removal is usually of minor importance^67^.

Overall, we speculate that dissimilative processes like denitrification have little ecological meaning here as they are constrained by low N availability in the water column or within sediments. Furthermore, the presence of both assimilative and dissimilative NO_3_^−^ reduction processes within the biofilm (see Fig. 3) suggests strong competition for N between denitrifiers and other bacteria/microalgae. As potential rates generally overestimate in situ rates due to high substrate availability, we argue that the biofilm tends to recycle and relocate N via coupled dissimilative and assimilative processes, avoiding permanent N losses and acting as a net N_2_ sink and as a source of particulate and dissolved N to the environment.

### Host excretion further releases N

As a matter of fact, we show significant DON release by the fiddler crab and associated microbiota (Fig. 5). This is an interesting finding since most of decapod crustaceans primarily excrete ammonia (NH_3_) or the conjugated acid NH_4_^+^^68^. Nevertheless, it has been shown that some decapods (e.g., shrimps living at lower temperatures than in our study site), can excrete DON, although this never exceeds NH_4_^+^ excretion^69^. In the fiddler crab, the significant amount of released DON might partly derive from fixed N which is not assimilated by the crab and its associated bacteria or microalgae. The biochemical mechanisms that promote such DON release should be addressed in future studies. Furthermore, it has rarely been questioned whether NH_4_^+^ excretion by animals is solely a physiological process or if it might also be attributed to invertebrate-bacteria associations^70^. For example, Samuiloviene et al.^38^ found active transcription of *nrfA* gene (encoding for DNRA) in tube dwelling chironomid larvae, suggesting the presence of active ammonifiers. In our study, the results from both qPCR and metagenomic analyses corroborate the presence of ammonifiers within the biofilm, which can potentially contribute up to 46% of net NH_4_^+^ production by the fiddler crab holobiont.

### The crab holobiont contributes to the ecosystem N budget

N_2_ fixation was one of the most dominant pathways compared to other N-cycling processes associated with fiddler crab microbiota, suggesting that this characteristic benthic invertebrate has a potentially relevant role in mangrove ecosystems as a vector of newly fixed N to the surrounding environment. The study area is a system where N_2_ fixation is an important conduit for bioavailable N^39^. Therefore, fixed N is an essential element in mangrove food webs, including detritus-feeding fiddler crabs. On the other hand, by selective grazing on bacteria or microalgae^15,71,72^ these crabs can redistribute or reduce N_2_ fixation in mangrove forests. Measured dissolved N production by the host, which likely comes from crab excretion, exudation of newly fixed N or its turnover within the biofilm, together with that associated to crab faeces—not measured here—can enrich with N the surrounding benthic system. Moreover, as the fiddler crabs intermolt and molt cycles last < 150 days^73^, the labile organic matter of the biofilm-covered carapace is delivered to the benthic system at least 3 times per year and can prime heterotrophy by relieving the very high C:N sediment ratios.

In a wider ecosystem context, considering a minimum abundance of 10 crab individuals per square meter^74^, fiddler crabs can produce 33 µmol m^−2^ d^−1^ of dissolved N which compensate/reverse dissolved N uptake measured at the sediment–water interface (− 71 µmol m^−2^ d ^−1^;^39^). In addition, N_2_ fixation associated with fiddler crabs can deliver 135 µmol N m^−2^ d^−1^ which compares to 27% of N_2_ fixation in surface microbial mats (500 µmol m^−2^ d^−1^;^39^). Unlike most bioturbating invertebrates, fiddler crabs temporally leave their burrows while feeding, mating or for territorial defence, moving to the surface sediment during low tide^24,75,76^. During high tide the crabs plug their burrows with sediment, residing either in a formed air chamber or crawling deeper to the flooded part of the burrow^23,77,78^. Because of crab respiration (~ 7 µmol O_2_ crab^−1^ h^−1^, data not shown) or oxidation of end-metabolic compounds (e.g., NH_4_^+^, H_2_S), oxygen is gradually consumed in the burrows^79^, promoting NO_3_^−^ reduction processes and transient accumulation of NH_4_^+^, despite these latter processes likely having little quantitative relevance at the ecosystem level.

## Conclusion

Mangroves ecosystems are increasingly challenged by anthropogenic activities, which may result in pressure to these ecosystems in terms of increasing nutrient or organic matter loading^80^. Under the pristine conditions of Cananéia region^39^, crab’s biofilm microbiota may be dominated by diazotrophic members, contributing fixed N to the environment, and possibly to the host. Conversely, if exposed to eutrophic conditions, fiddler crab holobionts may experience changes in the biofilm composition and metabolic repertoire, with a suppression of the energy-costly process of N_2_ fixation and an increase of dissimilative losses of excess N. Future studies should extend this combined molecular and biogeochemical approach to other study areas along environmental gradients, in order to verify to which degree fiddler crab carapace microbiota is environmentally assembled vs determined by host factors.

## Material and methods

### Study site

Specimens of the Atlantic mangrove fiddler crab (*Leptuca thayeri,* Rathbun 1900) were collected in their burrows during low tide from a muddy bank nearby a small channel (25°2′55.50″, 47°58′31.24″) located in the estuarine system of Cananéia, on the south coast of Brazil. During the sampling event surface (0.5 m depth) water temperature in the channel was 19.5 °C, salinity was 26.2 and dissolved oxygen concentration was 184.4 µM. This pristine estuarine system receives seasonally variable nutrient inputs depending on rainfalls, however, dissolved inorganic nitrogen (DIN) concentration in the system rarely exceeds > 4 µM of DIN^81^. The estuarine system, extending over an area of 110 km^2^, is part of the Cananéia–Iguape complex and is characterized by the presence of mangroves, restingas, inland seas and islands (Cananéia, Cardoso, Comprida, and Iguape). The region is part of the Cananéia–Iguape–Paranaguá Environmental Protection Area, and is recognized by UNESCO as part of the Biosphere Reserve. The estuarine system is connected to the South Atlantic Ocean by the Cananéia and Icapara inlets located, respectively, to the south and north of the system. Water circulation in the estuary channels is driven by a daily tidal flow and inflow of freshwater from continental drainage of several small rivers up to 6 m^3^ s^−1^ during dry season^82^. The intertidal stands are composed by *Spartina* meadows at the outermost portion, followed by *Laguncularia racemosa* in two stages of development. *Rhizophora* mangle followed by *Avicennia schauerianna* occupy the inner parts of the mangrove forests^83^.

### DNA extraction

Samples for DNA analysis were collected in the field from the carapace of randomly selected crabs (n = 3, with total surface area of ~ 8 cm^2^) by using swabs (after rinsing in 0.2-µm-filtered seawater), which were later preserved in RNAlater (Zymo Research). In the laboratory, DNA was extracted from three samples using the QIAamp Fast DNA Stool Mini Kit (QIAGEN). The lysis temperature was increased to 90 °C to improve the bacterial cell rupture. The final extracted DNA was subsampled for (1) 16S rRNA gene amplicon sequencing, (2) shotgun metagenomic sequencing, and (3) functional gene quantification by qPCR. Metagenome sequencing was carried out in one pooled sample (n = 3) to get sufficient amount of DNA for shotgun sequencing.

### 16S rRNA gene amplicon sequencing

16S rRNA gene sequences were amplified from extracted DNA using the primer pair Probio Uni and/Probio Rev, targeting the V3 region of the 16S rRNA gene sequence as described previously by Milani et al.^84^. High-throughput sequencing was performed at the DNA sequencing facility of GenProbio srl (www.genprobio.com) on an Illumina MiSeq with the length of 250 × 2 bp, according to the protocol previously reported in Milani et al.^84^. Sequencing yielded 144,418 paired-end reads (range 102,813–185,876) and the fastq data was analysed according to the DADA2 pipeline^85^ using the DADA2 1.12.1 package with R. Default settings were used with some exceptions, during quality trimming: truncLen = c(150,150), maxEE = 5, truncQ = 2, m, trimLeft = c(21, 22). This allowed to keep high quality reads and remove leftover primers from the sequences. FastQC 0.11.8 was used to manually check the quality of the trimmed reads^86^. During merging of the reads: minOverlap = 10, and during chimera removal: allowOneOff = TRUE, minFoldParentOverAbundance = 4. Sequences were classified against the SILVA 138 database^87^, and chloroplast sequences were removed. The final amplicon sequence variant (ASV) data was normalized as relative abundance (%). Shannon’s H alpha diversity was calculated in the software Explicet 2.10.15^88^ after sub-sampling to the lowest sample size (88,457 counts). A full list of all ASVs, taxonomic classifications and sequence counts are available in Supplementary Information 1.

### Shotgun metagenomic sequencing

Shotgun-based metagenomics analysis was performed on Illumina NextSeq with sequence length of 150 × 2 bp. Raw sequencing data consisted of 17.8 million paired-end reads and was manually checked for quality using FastQC^86^. Illumina adapters had been removed by the sequencing facility, and that no remains of PhiX control sequences were left was checked by mapping reads against the PhiX genome (NCBI Reference Sequence: NC_001422.1) using Bowtie2 2.3.4.3^89^. Reads were quality trimmed using Trimmomatic 0.36^90^ with the following parameters: LEADING:20 TRAILING:20 MINLEN:50. FastQC was used to check the quality of the trimmed reads. The trimmed data consisted of 17.4 million paired-end reads, with an average quality Phred score of 33 per base, and an average read length of 144 bp. Because low merging rate of the pairs, ~ 37% with PEAR 0.9.10^91^, the quality trimmed forward (R1) and reverse (R2) read-pairs were annotated separately. Protein annotation against the NCBI NR database was conducted by using the aligner Diamond 0.9.10^92^ in combination with BLASTX 2.6.0+^93^ with an e-value threshold of 0.001. The data was imported into the software MEGAN 6.15.2^94^ and analysed for taxonomy, using default lowest common ancestor (LCA) settings, with the NCBI accession numbers linked to NCBI taxa (MEGAN supplied database: prot_acc2tax-Nov2018X1.abin). Protein annotated sequences were analysed in MEGAN using the supplied database acc2interpro-June2018X.abin that links accession numbers to the InterPro database and Gene Ontology (GO) categories. The taxonomy and protein data attributed to N-cycling were extracted from MEGAN and the average read count for R1 and R2 was used for further analysis. In total, 9.0–9.2 million sequences were classified to taxonomy, and 4.9–5.0 million sequences to proteins (range of R1 and R2 data). To link taxonomy with protein classifications the “read name-to-taxonomy” and “read name-to-protein” tables were extracted from MEGAN using the inbuilt functions of the software. These tables were combined based on identical read names. A full list of taxonomy, related N-cycling proteins, and sequence counts are available in Supplementary Information 2.

### Functional gene quantification

Quantitative polymerase chain reactions (qPCR) were used to quantify the abundance of bacterial 16S rRNA gene and functional genes involved in N-cycling on the crab carapace: (1) genes of haem-containing nitrite reductase (*nirS*), (2) Cu-containing nitrite reductase (*nirK*), (3) bacterial and archaeal ammonia monooxygenase (*amoA*) and (4) cytochrome C nitrite reductase (*nrfA*). The primers and reference strains were used according to Samuiloviene et al.^38^. Quantitative PCR was performed with the StepOnePlus Real Time PCR system (ABI 7900 HT Sequence Detection System, PE Biosystems) using optical grade 96-well plates. The PCR reaction was run with a final volume of 20 µl containing 10 µl of SYBR Green master mix, 0.2 µM of forward and reverse primers, 2 mM of MgCl_2_ (25 mM) and 2 µl of DNA sample (diluted 1/10). The thermocycling conditions were as follows: 50 °C for 2 min; initial denaturation at 94 °C for 10 min; 40 cycles at 94 °C (1 min), 60 °C (1 min), 72 °C (1.5 min); and final elongation at 72 °C (5 min). To assess the specificity of amplifications, a melting curve analysis was performed in the range of 60–95 °C, with 0.3 °C increment. Each sample was analysed in triplicates. Triplicate no-template controls were also included in each qPCR assay. The abundance of target functional genes was expressed per number of 16S rRNA gene copy.

### Individual microcosm incubations

In the laboratory, collected fiddler crabs (n = 50) were left overnight in three aquaria (volume 20 l) with ambient water, continuous aeration and temperature control fixed at 19 °C for further experimental activities. Afterwards, single intermolt fiddler crab individuals were transferred into Plexiglas microcosms (n = 5, i.d. 4 cm, height ~ 20 cm, volume = 227 ± 3 ml) filled with unfiltered seawater from the sampling site. In parallel, control microcosms with water alone (n = 3) were prepared in order to correct process rates measured in crab microcosms. All microcosms were equipped with rotating magnets to ensure continuous water mixing (25 rpm) and were initially submersed, with the top open, in an incubation tank. Dark incubations started when microcosms top opening was closed with gas tight lids and lasted for < 6 h. At the beginning (from the incubation tank, in quadruplicate) and end of the incubations (from each microcosms) 50 ml aliquots were transferred to 12 ml exetainers (Labco Ltd) and fixed with 100 µl of 7 M ZnCl_2_ for N_2_:Ar measurements. In addition, two aliquots of 20 ml were collected, filtered (GF/F filters) and transferred into PE tubes and glass vials for inorganic and organic N analyses, respectively. Filtered water samples for spectrophotometric analyses were immediately frozen at − 20 °C until analysis (see details below). After incubation, crabs from all microcosms were used to determine carapace area, dry weight (at 60 °C for 48 h), and thereafter analysed for isotopic composition. The measured N excretion/production rates were normalized for the dry weigh (dw) crab biomass.

### Biofilm slurry incubations

^15^N tracer slurry incubations, which allow determination of potential rates, have commonly been used to investigate benthic N transformations^95^. The material for slurry incubation was collected from 22 crab carapaces (for a total area of ~ 60 cm^2^) by gentle brushing using a sterile toothbrush while holding single crab individual in separate glass beaker with 0.22-µm-filtered ambient water (500 ml). The final concentration of the scratched material, suspended in 500 ml water, corresponded to 28.39 ± 0.34 g_dw_ biofilm l^−1^ (at 60 °C for 48 h). The homogenized slurry, composed of material from carapace and 0.22-µm-filtered ambient water, was subsampled for oxic and anoxic incubations to measure different potential N metabolic pathways: (1) nitrification, (2) denitrification + DNRA, and (3) anammox. Potential nitrification rates were estimated by oxic incubation in Erlenmeyer flasks (n = 4) of 20 ml of the biofilm slurry enriched with ^14^NH_4_^+^ to a final concentration of 100 µM and maintained on table shaker at 19 °C. Water samples (5 ml) were collected at the beginning and at the end of the dark incubation, that lasted ~ 21 h. Samples were centrifuged, and the GF/F filtered supernatant was analysed for combined nitrite and nitrate concentrations (NO_x_^−^ = NO_2_^−^ + NO_3_^−^). Rates were expresses as NO_x_^−^ amount produced per individual crab, taking into account the mean dry weight of biofilm per carapace. Potential NO_3_^−^ reduction processes (denitrification, DNRA and anammox) were measured in anoxic incubations. For this incubation biofilm slurries were transferred into 12 ml exetainers (n = 24) without air bubbles and continuously suspended on a rotating shaker. An overnight preincubation was necessary to consume all dissolved oxygen and ^14^NO_3_^−^. Dissolved oxygen concentrations were monitored in additional 20 ml glass scintillation vials (n = 2) equipped with optical sensor spots (PyroScience GmbH). Thereafter, half of the exetainers was added with ^15^NO_3_^−^ to a final concentration of 100 µM whereas the remaining exetainers were added with ^15^NH_4_^+^  + ^14^NO_3_^−^ to a final concentration 100 µM. After the various additions, microbial activity was immediately terminated in three replicates of each treatment by adding 100 µl of 7 M ZnCl_2_. The other exetainers were maintained on the rotating shaker and incubated in the dark at 19 °C for 12 h. Every four hours three replicates from each treatment were retrieved and microbial activity terminated by adding 100 µl of 7 M ZnCl_2_. This was followed by determination of isotopic composition of produced ^15^N–N_2_ and ^15^N–NH_4_^+^ with the protocol explained below.

### Analytical procedures and rates calculation

Dissolved inorganic N concentrations were measured with a continuous flow analyser (Technicon AutoAnalyzer II, SEAL Analytical) using colorimetric methods^96^. NO_3_^−^ was calculated as the difference between NO_x_^−^ and NO_2_^−^. Dissolved NH_4_^+^ was analysed using the method by Treguer and Le Corre^94^. Net N_2_ fluxes were measured via the N_2_:Ar technique by membrane inlet mass spectrometry (MIMS) at Ferrara University (Bay Instruments^97^;) and corrected for Ar concentration and solubility based on incubation water temperature and salinity^98^. Isotopic samples for ^29^N_2_ and ^30^N_2_ production were analysed by gas chromatography-isotopic ratio mass spectrometry (GC-IRMS) at the University of Southern Denmark. Briefly, headspace subsamples were injected into a GC extraction line equipped with an ascarite trap, a Porapak R chromatographic column, a copper column heated to 600 °C, and a Mg(ClO_4_)_2_ trap^99^. The extraction line was coupled to an isotope ratio mass spectrometer (IRMS, Thermo Delta V Plus, Thermo Scientific) by means of a Conflo III interface. Samples for ^15^NH_4_^+^ production were analysed by the same GC-IRMS after conversion of NH_4_^+^ to N_2_^97^ by the addition of alkaline hypobromite^100^. Slopes of the linear regression of ^29^N_2_ and ^30^N_2_ concentration against time were used to calculate production rates of labelled N_2_–*p*^29^N_2_ and *p*^30^N_2_, respectively. Since *p*^29^N_2_ was not significant in ^15^NH_4_^+^  + ^14^NO_3_^−^ treatment, we deduced that anammox rate was negligible. Denitrification potential rate was calculated from the equations reported in the Thamdrup and Dalsgaard^101^. The slope of the linear regression of ^15^NH_4_^+^ concentration against time was used to calculate the production rate of labelled NH_4_^+^–*p*^15^NH_4_^+^. Potential DNRA rate was calculated according to Bonaglia et al.^102^. These NO_3_^−^ reduction rates were thus corrected for the actual ^15^N enrichment, and for individual specimen taking into account mean dry weight of biofilm per carapace.

Organic C and total N content and their isotopic composition in different crab tissues (~ 1 mg) were analyzed with a mass spectrometer (IRMS, Thermo Delta V, Thermo Scientific) coupled with elemental analyzer (ECS-4010, Costech Instruments) at the University of Sao Paulo. Before measurements samples were acidified with 1 M HCl to remove carbonates. C and N content was presented in percentage and their isotopic signatures were expressed in the form of δ ‰, according to the following equation:

$$\updelta = \, \left[ {{\text{R}}_{{{\text{sample}}}} /{\text{R}}_{{{\text{reference}}}} } \right) - 1] \times 1000$$

where R_sample_ is the isotopic ratio in the sample and R_reference_ is the isotopic ratio in the reference standard (Vienna Pee Dee Belminite (V-PDB) and atmospheric N_2_, respectively).

## Supplementary information

Supplementary Information 1

Supplementary Information 2

Supplementary Information 3

Supplementary Figure 1

## Data Availability

The raw sequence data supporting this study have been uploaded online and are available at the NCBI BioProject PRJNA549720.
