# Supplementary material for: N2 fixation dominates nitrogen cycling in a mangrove fiddler crab holobiont
Source: Sci Rep. 2020 Aug 18;10:13966. doi: 10.1038/s41598-020-70834-0 (PMC7435186; doi:10.1038/s41598-020-70834-0)
Supplement: Supplementary file 4 — Supplementary Figure 1 [file 41598_2020_70834_MOESM4_ESM.docx]

**N_2_ fixation dominates nitrogen cycling in a mangrove fiddler crab holobiont**

Mindaugas Zilius, Stefano Bonaglia, Elias Broman, Vitor Gonsalez Chiozzini, Aurelija Samuilovienė, Francisco J.A. Nascimento, Ulisse Cardini, Marco Bartoli


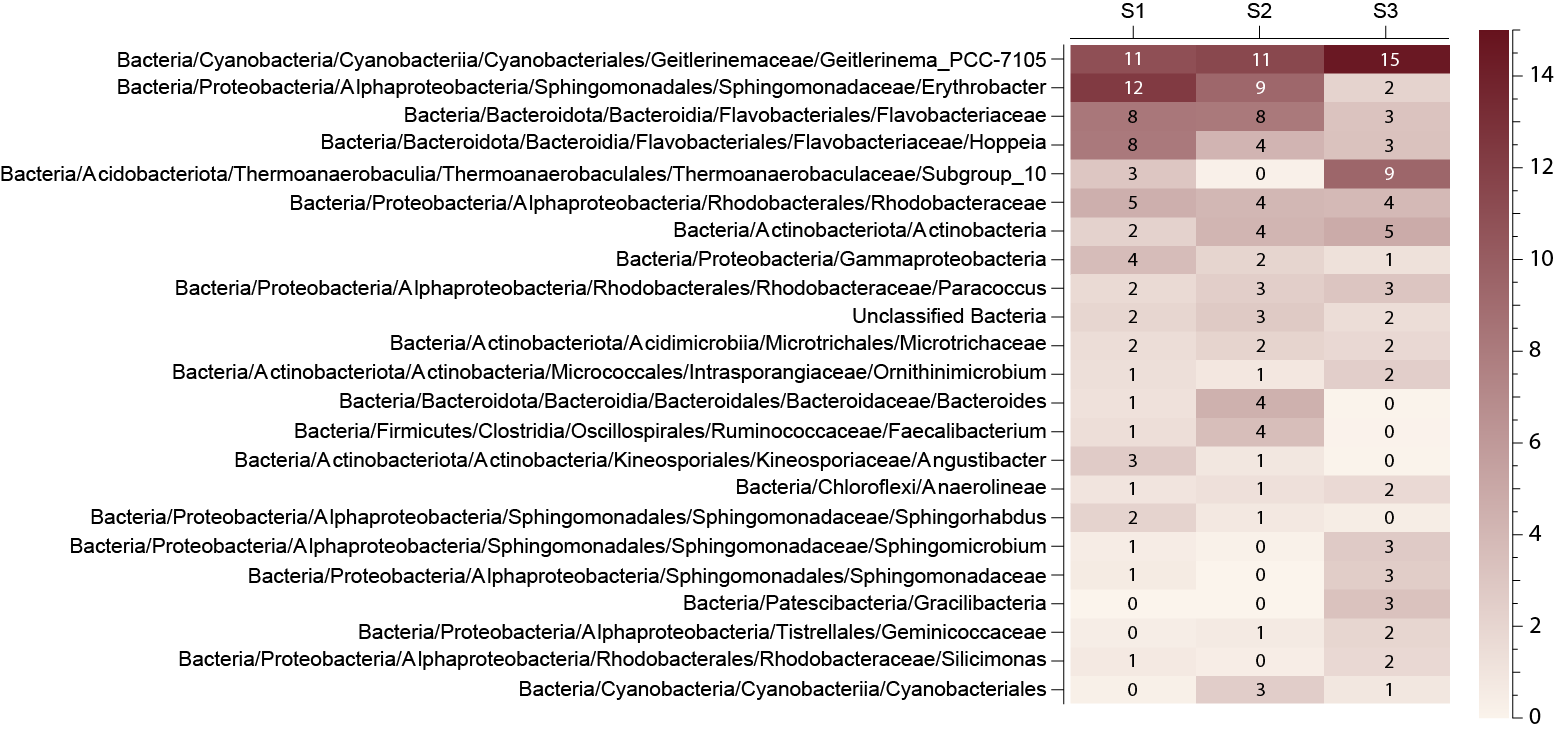


**Supplementary Figure 1.** Relative abundance of the top genera from the crabs’ carapace biofilm as obtained by 16S rRNA gene sequencing.
